# COVID-19, Inter-household Contact and Mental Well-Being Among Older Adults in the US and the UK

**DOI:** 10.3389/fsoc.2021.714626

**Published:** 2021-07-26

**Authors:** Yang Hu, Yue Qian

**Affiliations:** ^1^ Department of Sociology, Lancaster University, Lancaster, UK; ^2^ Department of Sociology, University of British Columbia, Vancouver, BC, Canada

**Keywords:** COVID-19, inter-household contact, mental health, older adults, virtual interaction

## Abstract

Interacting with family members and friends from other households is a key part of everyday life and is crucial to people’s mental well-being. The COVID-19 pandemic severely curtailed face-to-face contact between households, particularly for older adults (aged 60 and above), due to their high risk of developing severe illness if infected by COVID-19. In-person contact, where possible, was largely replaced by virtual interaction during the pandemic. This article examines how inter-household contact in face-to-face and virtual forms, as well as combinations of the two forms of contact, related to older adults’ mental well-being during the pandemic. Data from two national longitudinal surveys, collected from the same respondents before (2018–2019) and during (June 2020) the pandemic, were comparatively analysed: the Health and Retirement Study in the US and Understanding Society in the UK. The findings showed a notable increase in loneliness in the US and a decline in general mental well-being in the UK following the outbreak of COVID-19. In both countries, more frequent inter-household face-to-face contact during the pandemic was associated with better general mental well-being, but inter-household virtual contact, via means such as telephone and digital media, was not associated with general mental well-being in either the US or the UK. In the US, older adults who engaged more frequently in virtual contact were more likely to feel lonely during the pandemic, particularly if their face-to-face contact was limited. In both countries, the increase in loneliness following the outbreak of the pandemic was greater for older adults who reported more virtual contact. The findings suggest that household-centred crisis management during the COVID-19 pandemic had unintended mental health implications in both the US and the UK, despite contextual differences between the two countries. Although face-to-face contact between households helped to sustain older adults’ mental well-being, virtual contact was not a qualitatively equivalent alternative. The findings also provide an important evidence base for informing policy developments and for supporting the mental health of older people during the COVID-19 pandemic and in the longer term.

## Introduction

On 11 March 2020, the World Health Organization (2020) officially declared COVID-19 as a global pandemic. In most Western countries, public health and other policy responses to COVID-19 treated the household as a key unit of crisis management. Interventions such as home shielding, quarantine, and social distancing regarded the household as a principal social and resource unit, overlooking the importance of inter-household contact, i.e. everyday interaction with non-residential family members and friends (Willem et al., 2021). The threat posed by COVID-19 and its associated mitigation measures severely curtailed people’s inter-household in-person contact (Qian and Hanser, 2021). Older adults, defined as individuals aged 60 and above (United Nations, 2019), were more likely than their younger counterparts to practise home shielding and social distancing, partly because older people were known to be at a higher risk of becoming severely ill upon COVID-19 infection (Banerjee et al., 2020; Crimmins, 2020). A growing body of evidence points to a looming mental health crisis cascading from the COVID-19 pandemic (Pfefferbaum and North, 2020). Several studies have reported a sharp increase in mental distress, psychiatric disorders, and loneliness in the United States (US) and the United Kingdom (UK) during the pandemic (Daly et al., 2020; Groake et al., 2020; Killgore et al., 2020; Kumar and Nayar, 2020; Pierce et al., 2020; Niedwiedz et al., 2021). Inter-household contact has been identified as a key channel through which household-centred crisis management impacts mental well-being (Miller, 2020). The relationship between inter-household contact and mental well-being among older adults in the context of the COVID-19 pandemic has yet to be systematically analysed, a gap that this article aims to fill.

A long tradition of research on social networks and informal support in old age care has emphasised the importance of inter-household ties (Connidis and Barnett, 2018). The convoy model posits that social ties with non-residential family members and friends sustain care and resource exchanges, companionship, emotional support, and a sense of ontological security (Kahn and Antonucci, 1980; Fuller et al., 2020), all of which are essential to supporting older adults’ mental well-being (Rook et al., 2011). Furthermore, according to the stress-coping theory, the presence of a social convoy and the material and non-material support exchanged through social networks play a crucial role in mitigating generalised stress and stress related to specific life course processes and events such as ageing and illness (Wong and Ujimoto, 1998; Carver and Vargas 2011). However, despite the theoretical importance of inter-household ties in sustaining older adults’ mental well-being, relatively less attention has been paid to the state of inter-household contact and how it related to the mental well-being of older adults during the COVID-19 pandemic. Against this backdrop, the focus of this study on inter-household contact responds to researchers’ call for attention to routine “weak” ties beyond a focus on strong forms of material and care exchange (Sandstrom and Dunn, 2014).

Research before the pandemic showed that interaction with non-residential family members and friends enhanced older adults’ mental well-being (Becker et al., 2019). Inter-household contact often took the form of face-to-face meetings and activities, supplemented by virtual interactions via telephone or video calls, text messaging, and social media (Ignatow et al., 2013; Sandstrom and Dunn, 2014; Lomanowska and Guitton, 2016). When regular in-person contact was not viable, such as for transnational families and in disaster scenarios, individuals often resorted to virtual communication to maintain long-distance relationships with family members and friends, along with occasional in-person visits (Madianou and Miller, 2013; Hu et al., 2020).

As COVID-19 shaped face-to-face and virtual inter-household interactions in divergent ways, it is essential to distinguish the two forms of inter-household contact during the pandemic. On the one hand, scientific evidence that COVID-19 spreads via air and physical contact meant that face-to-face contact was severely curtailed through the implementation of lockdown, quarantine, home shielding, and social distancing measures (Chang et al., 2020; Lebow, 2020). On the other hand, virtual forms of contact, such as telephone calls and text messaging, were extensively used. In the first two decades of the 21st century, rapid technological advancements gave rise to increased and more diverse forms of virtual contact via digital means such as FaceTime, Zoom, and social media platforms (van Dijk, 2020). The trend of digitisation had accelerated during the pandemic, albeit unevenly across different demographic and socioeconomic groups (Chakravorti et al., 2020). It remains unclear whether and in what ways face-to-face and virtual forms of inter-household contact related to older adults’ mental well-being during the pandemic.

Although the psychological benefits of face-to-face contact for older adults were well established in research conducted before the COVID-19 pandemic (Becker et al., 2019), virtual contact may not be equally possible or beneficial. First, telephone calls and text messaging—the most common forms of virtual contact among older adults—are generally known to be insufficient in simulating face-to-face contact, partly due to their lack of visuality (Holtzman et al., 2017). Second, digital media use is dependent on access to the internet, device affordances, and technological know-how, which are often stratified along socioeconomic lines (Seifert et al., 2021). Third, intensive digital media use can cause stress or even burnout (Reinecke et al., 2017). Digital stress or avoidance tends to be greater among older people and those who are less tech-savvy (Reinecke et al., 2017). Whereas inter-household contact before the pandemic usually involved a blend of face-to-face and virtual interactions, virtual contact became the only viable means for many people to connect with non-residential family members and friends during COVID-19 (Arpino et al., 2021; Qian and Hanser, 2021). This study investigates whether, in the unprecedented context of the pandemic, virtual contact between households compensated for the lack of face-to-face contact in helping support older adults’ mental well-being.

Pre-pandemic social and demographic factors played a crucial role in conditioning inter-household contact during the pandemic. To explore potential contextual differences, this study comparatively assessed the situation in the US and that in the UK. These two countries were selected for analysis because they are characterised as having liberal welfare regimes (Esping-Andersen, 1990), although state welfare for older people tended to be more generous in the UK than in the US (Gaffney, 2016). In 2019, older adults (aged 60 and above) accounted for similar proportions of the populations in the US (23%) (US Census Bureau, 2021) and the UK (24%) (Office for National Statistics, 2019). On average, older adults in the US have more children than their UK counterparts, but the greater population dispersion in the US means that the former are less likely than the latter to co-reside with, or live close to, family members (Solé-Auró and Crimmins, 2014). As a result, face-to-face contact with non-residential family members before the pandemic may have been less frequent in the US than in the UK. In terms of virtual contact, levels of internet connectivity were similar among older adults in the two countries (67% in the US and 70% in the UK for those aged 65 and above) (Pew Research Center, 2017; Statista, 2020). However, as older adults in the US (compared with their counterparts in the UK) tend to live farther away from their families, they may have been more reliant on using the telephone, internet and digital media to keep in touch with non-residential family members.

In the first year of the pandemic (mid-March 2020–early 2021), the relationship between inter-household contact and mental well-being may have been re-configured differently in the US and the UK due to the two countries’ distinct responses to COVID-19. The UK government implemented two national lockdowns in 2020, which involved legally mandated household isolation, social distancing, the near-complete closure of hospitality and entertainment venues, and specific restrictions on inter-household mixing (Cabinet Office, 2021). According to the COVID-19 stringency index developed by Our World in Data (2021), stay-at-home requirements were implemented less strictly and on a state-by-state basis in the US, with no blanket policy of business closures. Given the UK’s more stringent enforcement of lockdown and social distancing measures, people in the UK were more likely than their US counterparts to be obliged by law to stay at home. Consequently, inter-household face-to-face contact during the first year of the pandemic would have been curtailed to a greater degree in the UK than in the US, making UK residents more reliant on virtual contact than their US counterparts. Due to the UK’s tighter restrictions on inter-household mixing, UK residents with restricted access to, and knowledge of, digital communication were more likely than their US counterparts to be limited in both face-to-face and virtual inter-household contact (Seifert et al., 2021). Given this possible pattern of “double exclusion”, this study adopts a holistic perspective and examines how face-to-face and virtual inter-household contact, and their distinct combinations, differentially related to older adults’ mental well-being in the US and the UK. It addresses three specific questions:

1. What was the status of older adults’ mental well-being during (vs. before) the COVID-19 pandemic in the US and the UK?
2. What were older adults’ patterns of inter-household contact during the pandemic in the US and the UK?
3. How did face-to-face and virtual forms of inter-household contact, as well as their distinct combinations, relate to older adults’ mental well-being during (vs. before) the pandemic?

To answer these questions, this study analysed high-quality data from two national longitudinal surveys conducted in the US and the UK, collected from the same respondents before (2018–2019) and during (June 2020) the COVID-19 pandemic. The study was designed to determine and compare how distinct forms of inter-household contact during the pandemic related to older adults’ mental well-being in the US and the UK. The findings were expected to illustrate the importance of inter-household contact in maintaining older adults’ mental well-being and to demonstrate the need to consider and address potential ramifications of household-centred pandemic responses for mental well-being among older adults. The findings were also expected to have implications beyond the immediate context of COVID-19. Rapid digitisation was already underway in many societies before the pandemic, and the pandemic considerably accelerated this process (Robinson, 2020). Against this backdrop, the study set out to illuminate the challenges and potential limitations of virtual communication in supporting the mental well-being of ageing populations.

## Data and Methods

### Data

The US data were drawn from the early release of the 2020 Health and Retirement Study (HRS) and the preceding main wave of the HRS in 2018. Initiated in 1992, the HRS is a nationally representative longitudinal survey of older adults in the US (Servais, 2010). In June 2020, the HRS collected information on respondents’ life circumstances during COVID-19, including their inter-household contact and mental well-being. Information on respondents’ socio-demographic characteristics and mental well-being was also collected in the main 2018 HRS. The UK data were obtained from the Understanding Society (USOC) COVID-19 survey and the preceding main waves of USOC. Initiated in 2009, USOC is a nationally representative longitudinal household survey. In June 2020, the USOC COVID-19 survey collected data on adults’ inter-household contact and mental well-being during the COVID-19 pandemic. Although further waves of the USOC COVID-19 survey are available, the analysis was limited to the June wave to ensure comparability with the US data. Comparable information about respondents’ mental well-being was also collected in the main USOC waves before COVID-19 in 2018–2019.

Following a panel design, the HRS and USOC data can be used to trace changes in the respondents’ mental well-being from before to during the pandemic. Information on face-to-face and virtual contact during the pandemic was collected in both waves of the HRS, but it was not collected in the pre-pandemic wave of USOC. Therefore, to ensure cross-national comparability, only measures for inter-household contact during the pandemic were used in our analysis. The HRS and USOC data had to be carefully interpreted with reference to their different survey modes. The 2020 HRS survey was administered via postal paper questionnaires, while the USOC COVID-19 survey took the form of online self-completion questionnaires. The survey weights provided by the HRS and USOC teams were used in all of the analyses to ensure that the results were representative of the US and UK populations, respectively. But the datasets may have under-represented 1) homeless and institutionalised populations in both countries, 2) those with limited internet access in the UK, and 3) those with disabilities or who were severely ill with COVID-19.

### Analytical Sample

Following the United Nations’ (2019) definition of older adults, the analytical sample was first limited to respondents aged 60 and above at the time of the 2020 (COVID-19) surveys (*N*_HRS_ = 1,736 respondents, *N*_USOC_ = 5,472 respondents). Next, the sample was restricted to COVID-19 survey respondents with valid records in the preceding main waves of the HRS and USOC (*N*_HRS_ = 1,623, *N*_USOC_ = 5,311). The data from the preceding main waves were then merged with those from the COVID-19 waves. Finally, listwise deletion was applied to cases with missing or invalid values for the variables used in the analysis, yielding a final analytical sample of 1,391 US respondents and 5,148 UK respondents. Each respondent was observed twice—before and during the pandemic. Little’s test indicated that the values were missing at random (Li, 2013). Step-by-step information on the sample construction is provided in Supplementary Table S1.

### Dependent Variables

#### General Mental Well-Being

Both before and during the pandemic, the HRS captured the respondents’ general mental well-being using the eight-item Center for Epidemiologic Studies Depression (CES-D) scale (Steffick, 2000). The respondents were asked whether in the week before the survey they had felt 1) depressed, 2) that everything was an effort, 3) that sleep was restless, 4) happy, 5) lonely, 6) that they enjoyed life, 7) sad, and 8) that they could not get going. The response to each item was recorded using a dummy variable, with 1 indicating that the respondent had experienced the feeling specified in the item and 0 otherwise. Items 4) and 6) were reverse-coded such that a value of 1 indicated poor mental well-being across the board. As the eight items exhibited a high level of internal consistency (Cronbach’s alpha = 0.79 both before and during COVID-19), their scores were summed to create a composite scale of general mental well-being. This scale ranged from 0 to 8, with higher scores indicating poorer mental well-being.

USOC measured the respondents’ general mental well-being both before and during COVID-19, using the 12-item General Health Questionnaire (GHQ-12) (for questionnaire wording and measure validity, see El-Metwally et al., 2018). Despite slight differences, the GHQ-12 is broadly comparable with the CES-D. Most of the CES-D items, such as depression, sleeplessness, enjoyment of daily activities, general happiness, and ability to face problems, are also included in the GHQ-12. To make the HRS and USOC measures more comparable, caseness scores were adopted to record the responses to each GHQ-12 item using a dummy variable akin to that for the CES-D. The GHQ-12 caseness scale ranged from 0 to 12, with higher scores indicating poorer mental well-being. The scale had a high level of internal consistency (Cronbach’s alpha > 0.90 both before and during COVID-19).

An additional variable was generated to capture the difference in the respondents’ general mental well-being before and during COVID-19 by subtracting each respondent’s pre-pandemic general mental well-being score from the corresponding score during the pandemic. For this change-score variable, a positive value indicated a decline, whereas a negative value indicated an improvement in mental well-being. Because the mental well-being scales and change scores had different ranges in the US and the UK, they were standardised within each country and the standardised scores were used in all models to facilitate cross-national comparisons. Although general mental well-being measures such as the CES-D and GHQ-12 are more susceptible to false positives than clinical measures (Coyne et al., 1991), the goal of this study was not to make clinical diagnoses. The CES-D and GHQ-12 scales have been shown to adequately capture both between-person differences and within-person changes in mental well-being (Steffick, 2000; El-Metwally et al., 2018).

#### Perceived Loneliness

Our second set of dependent variables measured the respondents’ feelings of loneliness as an important and specific dimension of mental well-being. Both the HRS and USOC captured the extent to which the respondents felt lonely (since the pandemic for the HRS and in the last four weeks for USOC), using three response categories: 1) never/hardly ever, 2) sometimes, and 3) often. Although the HRS and USOC loneliness measures covered slightly different time periods, both captured the respondents’ feelings of loneliness during the pandemic. The HRS further asked the respondents to compare their feelings of loneliness during and before COVID-19, and recorded the responses using three categories: 1) no change, 2) less lonely, and 3) lonelier. Although USOC did not ask the respondents to compare their feelings of loneliness during and before COVID-19, the main USOC waves collected comparable data on the respondents’ perceived loneliness before COVID-19. By comparing each respondent’s responses before and during the pandemic, a variable was generated to capture changes in the USOC respondents’ loneliness using the same measurement scheme as the HRS (i.e. no change, less lonely, and lonelier).

### Key Predictors: Inter-household Contact During the Pandemic

#### Face-To-Face Contact

The HRS asked its respondents how often they met up with non-residential children, other family members, and friends. Both pre-arranged and chance meetings were included. The response categories captured the frequency of contact, and they were reverse-coded to range from 1, least frequent (never or less than once a year), to 6, most frequent (more than three times a week). The three HRS items for face-to-face contact had a moderate level of internal consistency (Cronbach’s alpha = 0.53). The scores for the three items were added up to create a composite scale, with higher values indicating more frequent face-to-face contact. In USOC, a single measure was used to capture face-to-face contact with non-residential family members and friends. The responses were recorded on a 7-point Likert type scale, which were reverse-coded to range from 1, least frequent (never), to 7, most frequent (daily).

#### Virtual Contact

The HRS used nine items to measure how often the respondents interacted with non-residential children, other family members, and friends via telephone, email, and social media (Skype, Facebook, and other platforms), respectively. The response categories were the same as those for face-to-face contact. The items had a high level of internal consistency (Cronbach’s alpha = 0.81). All of these items were combined into one virtual contact scale by summing their scores, with higher scores indicating more frequent virtual contact. The USOC COVID-19 survey used two similar measures to capture textual (text messaging and email) and audio/video (telephone, FaceTime, Zoom) interactions with non-residential family members and friends in the four weeks before the survey, using the same response categories as those for face-to-face contact. The two measures were combined (Cronbach’s alpha = 0.76) into a single scale by adding up their scores, with higher scores indicating more frequent virtual contact. Because USOC captured telephone and digital contact using a single measure, it was not possible to further distinguish the two forms of virtual contact.

#### Imputation of Missing Values

To minimise sample loss for the HRS, missing values were imputed using a technique adopted by Abdelhadi and England (2019). Within a cluster of the same form of contact (face-to-face, telephone, email, or social media) with children, other family members, and friends, if a respondent had a missing value for one item, a regression-based prediction was produced estimating their response to the missing item based on their valid responses to the other two items. Cases with missing values for two or all of the contact items within each cluster were deleted. Supplementary analyses using listwise deletion without imputation yielded similar results to those reported in this article. To facilitate cross-national comparison, the inter-household contact scales were standardised within the US and the UK, respectively.

### Control Variables

As shown in Table 1, the list of control variables covers a range of factors potentially associated with mental well-being and inter-household contact. They included the respondents’ age at the time of the COVID-19 surveys and their gender. As ethnic/racial minority groups faced particular health and economic risks during the pandemic (Hu, 2020; Patel et al., 2020), a dummy variable was used to distinguish ethnic/racial minority status. In the US, ethnic/racial minority groups were distinguished from non-Hispanic whites (US Census Bureau, 2021). In the UK, ethnic minority groups included respondents who did not self-identify as white British, Irish, or European (Office for National Statistics, 2021). Immigrant status (i.e. not born in the country) was controlled for in both countries. The education variable distinguished whether a respondent had obtained a higher education degree, i.e. college education or above (Qian and Hu, 2021).

**TABLE 1:** Sample characteristics.

	US	UK
Variables	Mean/proportion	Mean/proportion
Age during COVID-19 (range: 60–99)	70.72 (8.54)	70.28 (6.61)
Female (ref. = male)	0.54	0.52
Ethnic/racial minority (ref. = no)	0.18	0.03
Migrant (ref. = non-migrant)	0.08	0.05
Higher education degree (ref. = no)	0.42	0.35
Living alone (ref. = no)	0.25	0.26
Working during COVID-19 (ref. = no)	0.28	0.23
Had/has COVID-19 (ref. = no)	0.02	0.02
Self-rated health during COVID-19 (range: 1–5)	3.25 (0.96)	3.04 (0.96)
Satisfaction with household income (range: 1–5)	3.62 (1.07)	3.75 (1.00)
*N* (respondents)	1,391	5,148

*Notes*: Ref. = reference category, which is coded as 0 for dummy variables. Mean values reported for continuous variables and proportions reported for dummy variables. Standard deviations in brackets.

Moreover, solo living was captured using a dummy variable, as individuals living alone may have distinct inter-household dynamics. Respondents who worked were distinguished from those who did not work during the pandemic, to account for potential differences in their social interactions. A dummy variable was used to capture whether the respondents had contracted COVID-19. Individuals’ mental well-being is positively associated with their (self-rated) health (Levinson and Kaplan, 2014). In both the HRS and USOC, the respondents’ self-reported health during COVID-19 was measured using a 5-point Likert scale, which was reverse-coded to range from 1 (poor) to 5 (excellent). Finally, the pandemic placed significant economic strain on many people (Hu, 2020), which may have spilt over to undermine their mental well-being. Thus, respondents’ self-reported satisfaction with their household income was included as a control variable. Given that the response categories for this measure ranged from 1 (not satisfied at all) to 5 (completely satisfied) for the HRS but from 1 (completely dissatisfied) to 7 (completely satisfied) for USOC, the USOC measure was rescaled to a range of 1 to 5, in line with the HRS measure.

In supplementary analyses, additional control variables were tested, including age squared, household size, number of children, changes in work status, and housing tenure. These variables were not associated with the respondents’ mental well-being or its changes. Including them neither affected the key results for inter-household contact nor improved the overall model fit. Particularly given the relatively small size of the US sample, these variables were excluded for parsimony and to ensure sufficient statistical power for the models. Mental health research in gerontology often controlled for functional limitations and chronic conditions (such as high blood pressure and diabetes) (Heine et al., 2019). However, these measures neither contributed to the overall model fit nor affected the key results for inter-household contact, partly because their effects had already been captured by the variable indicating self-reported health. Self-rated health was included in our final analysis because it was more consistently measured across the two countries than functional limitations and chronic conditions. Respondents’ marital and partnership status was not included in our models, because it was highly collinear with the living alone dummy. Due to the large number of missing values for individual and household income, income was not included in the analysis, but it was partly accounted for by the financial satisfaction measure. Supplementary analysis that controlled for income based on the respondents without missing values yielded consistent results. COVID-19 related concerns, measured only in the HRS, were negatively associated with the respondents’ mental well-being. However, since these concerns were not measured in USOC and they did not affect the association between inter-household contact and older adults’ mental well-being in the US, they were not included as covariates.

### Analytic Strategy

The analysis was carried out in two steps. First, descriptive statistics were used to delineate patterns of mental well-being during (vs. before) COVID-19, as well as patterns of inter-household contact during the pandemic. Next, regression models were fitted to examine how inter-household contact related to older adults’ mental well-being during COVID-19 and its changes relative to the pre-pandemic era. Ordinary least squares regressions were used to model general mental well-being and its change scores, as the residual distributions were within a range sufficient to assume a normal distribution. Ordinal logit regressions were used to model loneliness during COVID-19 and multinomial logit regressions were used to model changes in loneliness.

The models were fitted in two stages. The main effects of inter-household face-to-face contact and virtual contact were estimated first, followed by the interaction effects of the two. Following the best practices for interpreting and presenting interaction effects recommended by Mize (2019), predicted values of general mental well-being and loneliness were calculated based on distinct combinations of inter-household face-to-face and virtual contact. Compared with a mere focus on the statistical significance of interaction terms, this approach provided a more intuitive and substantively relevant interpretation of interaction effects. The HRS and USOC samples were analysed separately, partly because of slight cross-national differences in some of the original survey instruments. This means that the US and UK results were substantively but not statistically comparable. Given that both the HRS and USOC are household panel surveys, robust standard errors were estimated to account for sample clustering at the household level (Hoechle, 2007).

## Results

### Descriptive Results

**FIGURE 1:**
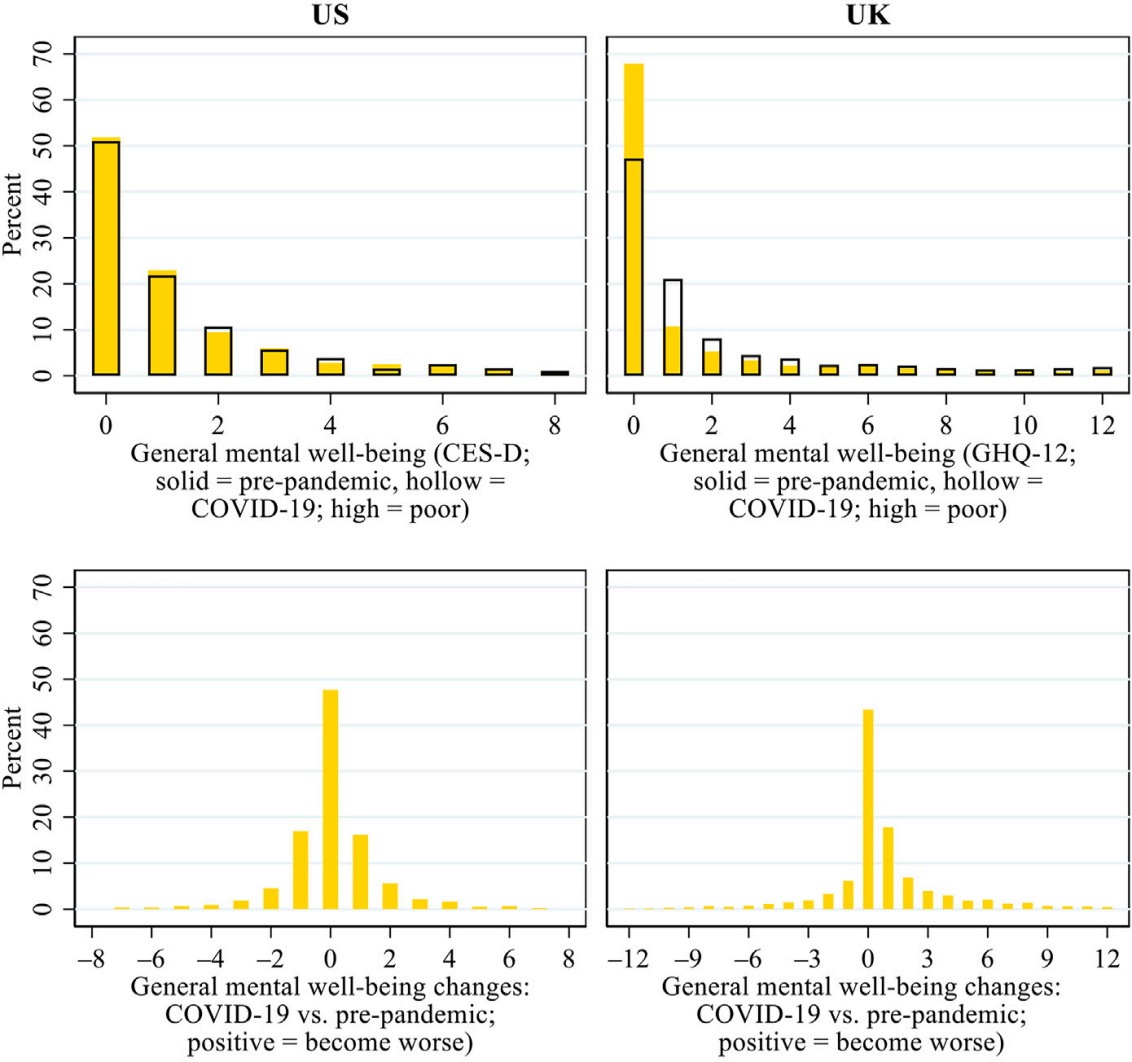
Patterns of general mental well-being during COVID-19 and its changes from before the pandemic in the US and the UK.*Note*: CES-D, Centre for Epidemiologic Studies Depression scale; GHQ, General Health Questionnaire.

Figure 1 presents the patterns of older adults’ general mental well-being during COVID-19 and its changes relative to the pre-pandemic era. In the US, more than half of the respondents scored 0 (indicating the best possible mental well-being) both before and during COVID-19. There was no overall decline in general mental well-being during the pandemic in the US, as similar proportions of the US respondents experienced an improvement (26.4%) and a decline (24.5%) in mental well-being. In the UK, the proportion of respondents who scored 0 on the general mental well-being scale decreased from 62.6% before COVID-19 to 46.3% during the pandemic. The results for the change scores show that a larger proportion of the UK respondents experienced a decline (37.6%) than an improvement (20.3%) in general mental well-being. Together, these results suggest that the pandemic and its associated public health and policy responses undermined older adults’ general mental well-being in the UK but not in the US.

**FIGURE 2:**
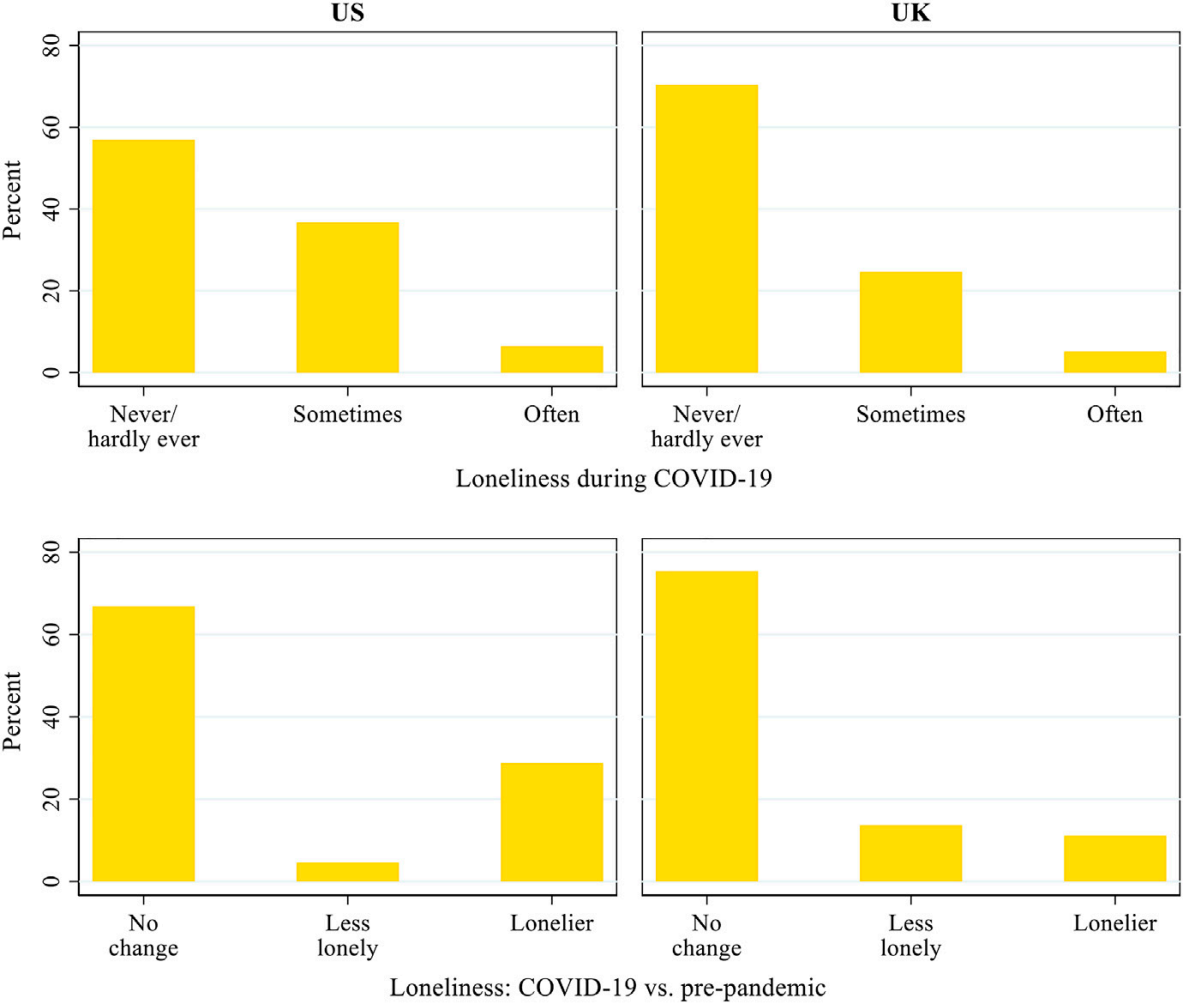
Patterns of loneliness during COVID-19 and its changes from before the pandemic in the US and the UK.

Figure 2 describes the prevalence of loneliness during the pandemic and its changes relative to before COVID-19. The results show that during COVID-19, a larger proportion of the US respondents reported feeling lonely than their UK counterparts did. In the US, 36.7 and 6.4% of the respondents sometimes felt lonely and often felt lonely, respectively, compared with 24.6 and 5.1% in the UK. Compared with before the pandemic, 4.6% of the US respondents became less lonely, whereas 28.7% became lonelier during the pandemic. In the UK, similar proportions of respondents became less lonely (13.6%) and lonelier (11.1%). Therefore, the negative impact of the pandemic and its associated migitation measures on older adults’ loneliness appears to have been greater in the US than in the UK.

**FIGURE 3:**
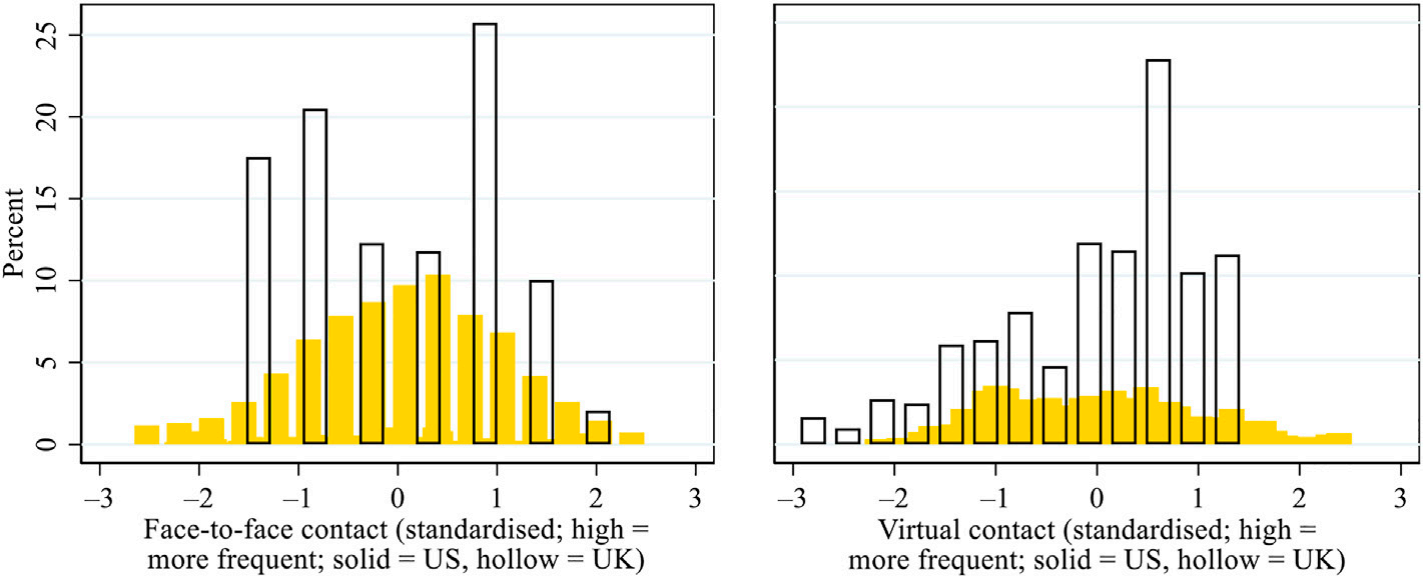
Patterns of inter-household face-to-face and virtual contact during the COVID-19 pandemic in the US and the UK. *Note*: Given that different measures were used in the US and the UK for inter-household contact, we standardised the scores within each country to facilitate cross-national comparisons.

Figure 3 presents the patterns of inter-household face-to-face and virtual contact during the pandemic, based on standardised scores. In the US, both forms of contact were normally distributed. In the UK, however, the respondents tended to report infrequent face-to-face contact but frequent virtual contact during the pandemic, which can be attributed to two possible reasons. First, the US respondents were surveyed using paper questionnaires, whereas the UK respondents were surveyed online, suggesting that the latter may have had greater digital access, capacity, and/or know-how and have been less restricted in their digital communication than the former. Second, as lockdown and household-centred pandemic responses were more stringently implemented in the UK than in the US, inter-household face-to-face contact may have been curtailed to a greater degree in the UK than in the US, whereas older adults in the UK may have been more dependent on virtual contact than their US counterparts.

### Regression Results: Main Effects of Inter-household Face-To-Face and Virtual Contact

**FIGURE 4:**
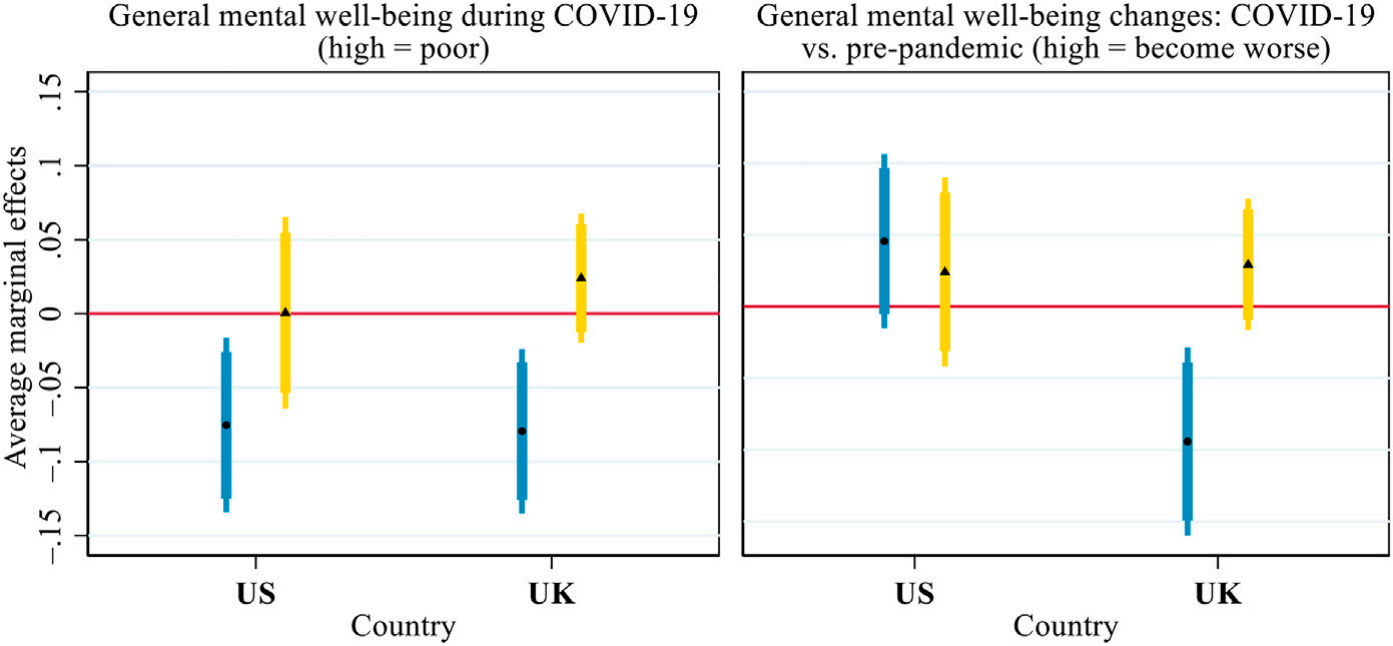
Average marginal effects of inter-household face-to-face (round dot, blue bar) and virtual (triangle, yellow bar) contact on general mental well-being during COVID-19 and its changes from before the pandemic. Notes: Both general mental well-being and its change scores were standardised within each country to facilitate the comparison between the US and the UK. Thick error bars indicate 90% confidence intervals and thin error bars indicate 95% confidence intervals. Calculations based on the models presented in Supplementary Table S2, controlling for all covariates presented in Table 1.

Figure 4 presents the average marginal effects (AMEs) of inter-household face-to-face and virtual contact (i.e. the effects of a one standard deviation change in the contact measures) on general mental well-being. As lower scores indicated better mental well-being during COVID-19 or an improvement in mental well-being relative to before COVID-19, a negative AME denoted the mental well-being benefit associated with inter-household contact. In the US, more frequent inter-household face-to-face contact was associated with better general mental well-being during the pandemic (AME = –0.075, *p* < 0.05), but it was not associated with changes in general mental well-being from before to during the pandemic. In the UK, frequent face-to-face contact was associated with both better general mental well-being during COVID-19 (AME = –0.079, *p <* 0.01) and a smaller mental well-being decline after the outbreak of the pandemic (AME = –0.094, *p <* 0.01). In both countries, however, inter-household virtual contact was associated with neither general mental well-being during the pandemic nor the difference in general mental well-being before vs. during the pandemic.

**FIGURE 5:**
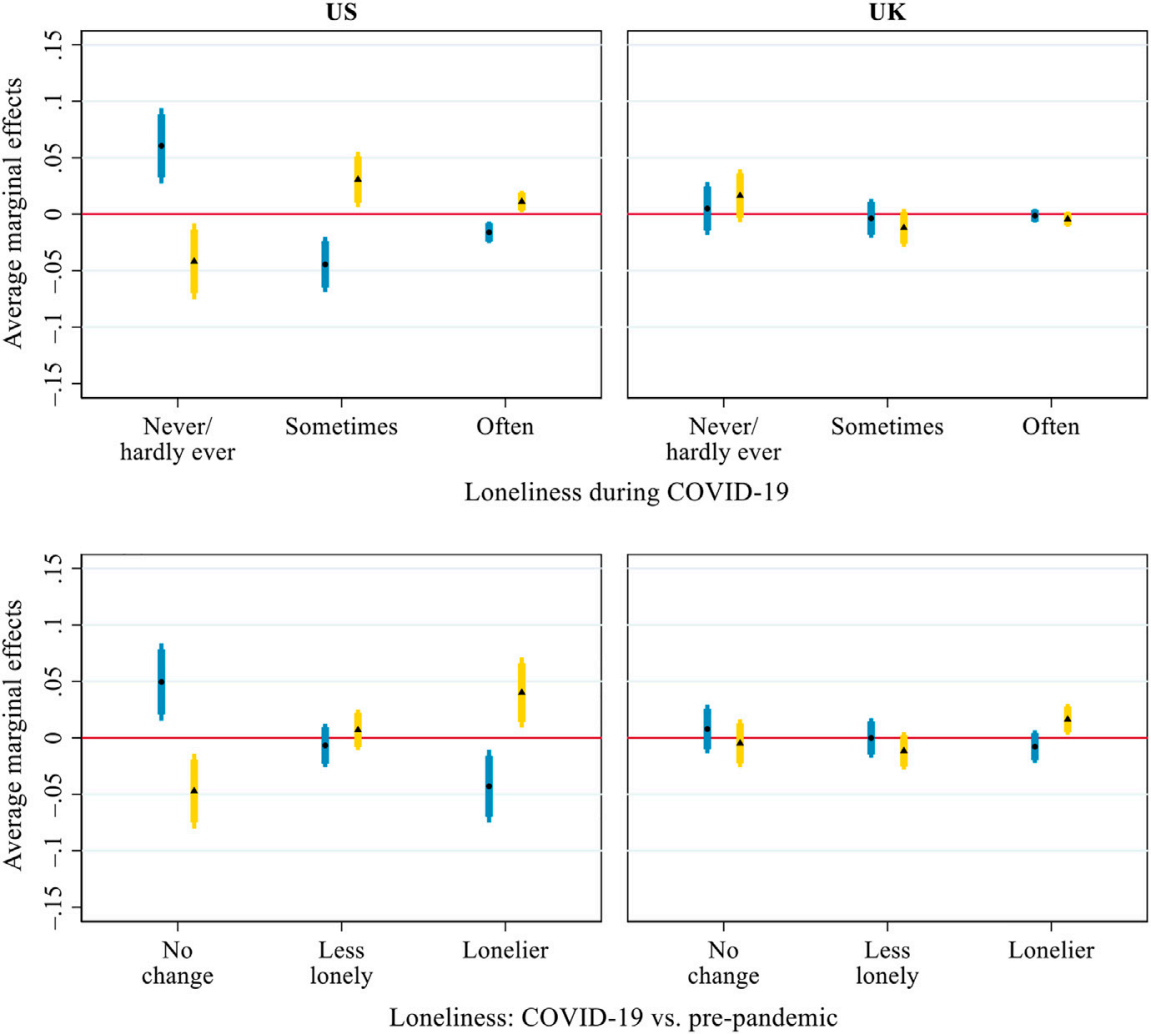
Average marginal effects of inter-household face-to-face (round dot, blue bar) and virtual (triangle, yellow bar) contact on loneliness during COVID-19 and its changes from before the pandemic. Notes: Thick error bars indicate 90% confidence intervals and thin error bars indicate 95% confidence intervals. Calculations based on the models presented in Supplementary Table S3, controlling for all covariates presented in Table 1.

Figure 5 presents the AMEs of inter-household face-to-face and virtual contact on older adults’ perceived loneliness. US respondents with more frequent face-to-face contact were less likely to sometimes feel lonely (AME = –0.044, *p <* 0.001) and often feel lonely (AME = –0.016, *p <* 0.01) during the pandemic and less likely to have become lonelier than before (AME = –0.043, *p <* 0.01). For virtual contact, the opposite pattern was observed. US respondents with more frequent virtual contact were more likely to sometimes feel lonely (AME = 0.031, *p <* 0.05) and often felt lonely (AME = 0.011, *p <* 0.05) during the pandemic. They were also more likely to have become lonelier than before the pandemic (AME = 0.040, *p <* 0.05). In the UK, however, face-to-face contact and virtual contact were not associated with older adults’ loneliness during the pandemic or its changes from before the pandemic, with only one exception: UK respondents with more frequent virtual contact were more likely to have become lonelier during the pandemic than before (AME = 0.016, *p* < 0.05).

### Regression Results: Interaction Effects of Inter-household Face-To-Face and Virtual Contact

**FIGURE 6:**
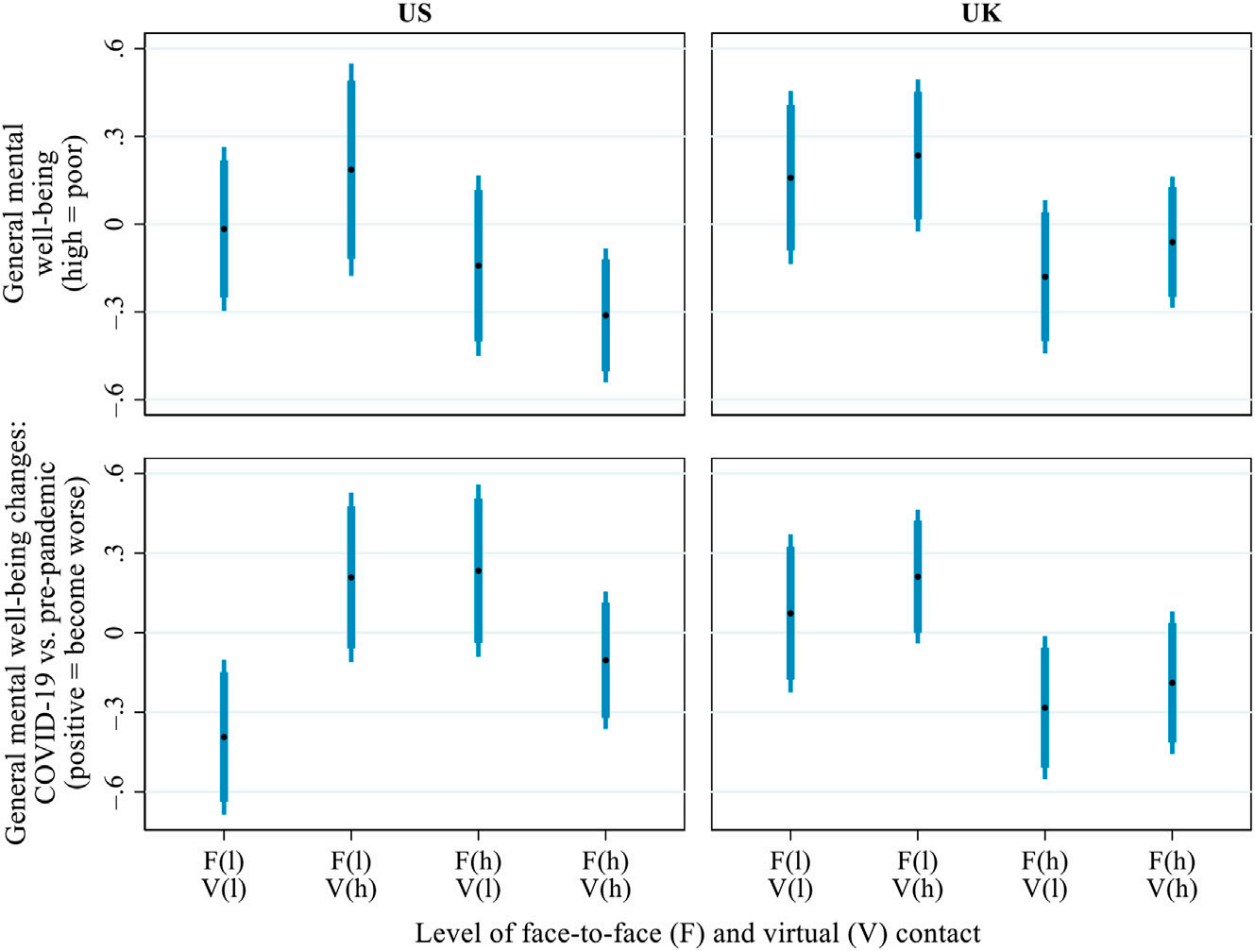
Predicted values of general mental well-being (high = poor) during COVID-19 and its changes from before the pandemic (positive = become worse), by the interaction of inter-household face-to-face and virtual contact. Notes: Both mental well-being and its change scores were standardised within each country for the ease of comparison between the US and the UK. Thick error bars indicate 90% confidence intervals and thin error bars indicate 95% confidence intervals. In the brackets on the x-axis, ‘l' = low level (i.e. 2 standard deviations below the mean) and ‘h' = high level (i.e. 2 standard deviations above the mean). Predictions based on the models presented in Supplementary Table S4, controlling for all covariates presented in Table 1.

Figure 6 presents the predicted values for general mental well-being during COVID-19 and its changes from before the pandemic across distinct combinations of inter-household face-to-face contact and virtual contact during the pandemic (cf. Mize, 2019): 1) infrequent contact in both forms, 2) infrequent face-to-face contact but frequent virtual contact, 3) frequent face-to-face contact but infrequent virtual contact, and 4) frequent contact in both forms. When calculating the predictions, “frequent” and “infrequent” were defined as two standard deviations above and below the national mean, respectively.

In the US, variations in the respondents’ general mental well-being across the four profiles of inter-household contact were not statistically significant, partly due to the small sample size. In general, however, those with infrequent face-to-face but frequent virtual contact tended to have the poorest mental well-being during the pandemic, and those with high levels of both face-to-face and virtual contact had the best general mental well-being. Interestingly, older adults in the US who were doubly excluded from both face-to-face and virtual inter-household contact experienced a small mental well-being improvement. This counter-intuitive result may be because some of the respondents pro-actively withdrew from inter-household contact to minimise the risk of COVID-19 infection. In turn, the enhanced perceived health safety may have benefitted their mental well-being. However, to fully test this conjecture, it would have been necessary to capture the respondents’ pre-pandemic inter-household contact as well as their subjective perceptions of risks related to inter-household contact during the pandemic. But no such data were available. In the UK, no notable interaction was found between the two forms of inter-household contact: irrespective of the frequency of virtual contact, frequent face-to-face contact was associated with better general mental well-being during COVID-19 and a smaller mental well-being decline relative to the pre-pandemic era.

**FIGURE 7:**
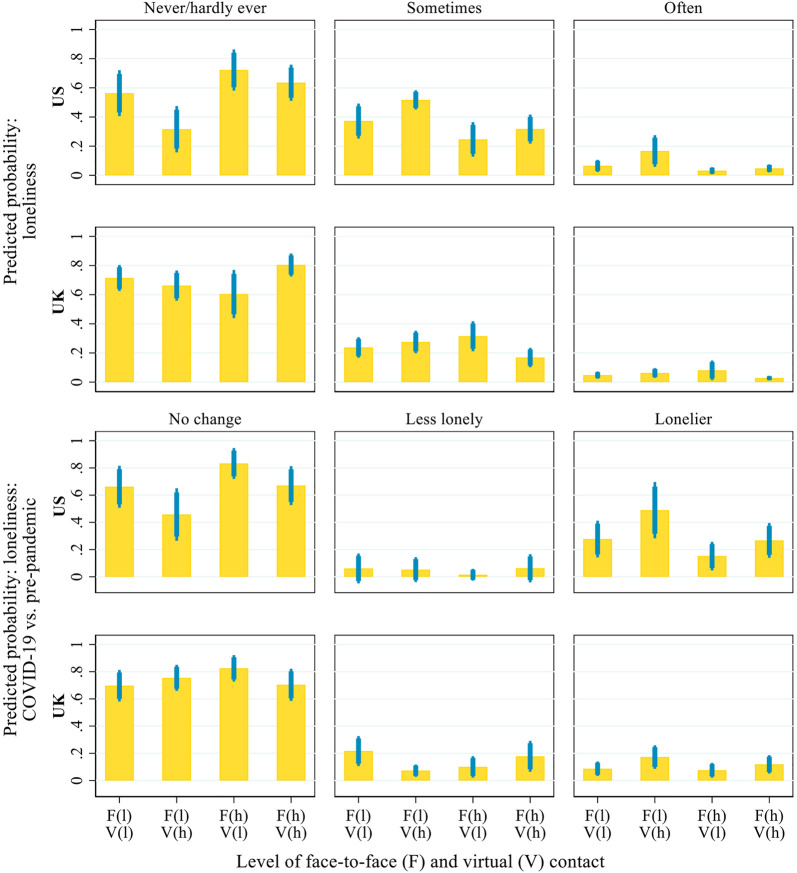
Predicted probability of loneliness during the COVID-19 pandemic and its changes from before the pandemic, by the interaction of inter-household face-to-face and virtual contact. Notes: Thick error bars indicate 90% confidence intervals and thin error bars indicate 95% confidence intervals. In the brackets on the x-axis, ‘l' = low level (i.e. 2 standard deviations below the mean) and ‘h' = high level (i.e. 2 standard deviations above the mean). Predictions based on the models presented in Supplementary Table S5, controlling for all covariates presented in Table 1.

Figure 7 presents the predicted probability of loneliness during the pandemic and its changes relative to before the pandemic across the same four profiles of inter-household contact (defined as shown in Figure 6). The results indicate that US respondents with frequent virtual but infrequent face-to-face contact were the most likely to feel lonely during the pandemic and to have become lonelier than before the pandemic, compared with those in the other three profiles. Specifically, only 31.6% of the US respondents with frequent virtual but infrequent face-to-face contact did not feel lonely during the pandemic, compared with 56.3–72.2% in the other three groups. About 49% of the US respondents with frequent virtual contact but infrequent face-to-face contact became lonelier after the outbreak of the pandemic, compared with 15.2–27.7% in the other groups. Thus, the results suggest the limited role of virtual contact in mitigating older adults’ loneliness in the US or else that older adults who felt lonely were particularly likely to initiate virtual inter-household contact. In the UK, respondents who had frequent inter-household contact in both face-to-face and virtual forms during the pandemic were the least likely to feel lonely: about 80.4% of the UK respondents with high levels of both virtual and face-to-face contact reported that they never or hardly ever felt lonely during the pandemic, compared with 60.4–71.4% in the other three groups.

### Results for Control Variables

Although the focus of this study was on the relationship between inter-household contact and mental well-being, our analysis also revealed how older adults’ mental well-being and its changes varied according to their socio-demographic characteristics (see online supplementary tables for full results). The results for our control variables were largely in line with the emerging evidence on socio-demographic variations in the mental well-being impact of the pandemic (Daly et al., 2020; Killgore et al., 2020; Kumar and Nayar, 2020; Luchetti et al., 2020; Pierce et al., 2020). For example, in both the US and the UK, older adults living alone, women (as compared to men), those with poorer self-reported health, and those with less financial satisfaction were more likely to exhibit poorer general mental well-being and feel lonely during the pandemic. COVID-19 infection adversely impacted older adults’ general mental well-being in the UK but not in the US. Older women and older adults living alone in both countries, those with less financial satisfaction in the US, and those with poorer self-reported health in the UK were more likely to report that they had become lonelier after the outbreak of the pandemic than before it. Although research has indicated the socioeconomic and health vulnerabilities of racial/ethnic minority groups during the pandemic (Hu, 2020; Patel et al., 2020), no statistically significant disparities were found in older adults’ general mental well-being or loneliness by racial/ethnic status. This finding may have been because racial/ethnic minority groups were more likely to co-reside with extended family members during the pandemic (Nafilyan et al., 2021) and were thus less likely to suffer from loneliness.

### Robustness Checks

To ensure the robustness of our results, a series of sensitivity analyses were conducted using alternative measures and alternative modelling strategies. First, it was not possible to capture a decline in mental well-being among those already reporting the poorest mental well-being before the pandemic. Similarly, there was little room for improvement among those with the best mental well-being before the pandemic. Therefore, a sensitivity analysis was conducted to examine whether the results from the mental well-being change-score models were affected by these “floor” and “ceiling” effects. When mental well-being scores collected before the pandemic were included in the models, the results differed little from those reported in this article. Second, the measures of general mental well-being and loneliness used in the main analysis were selected to ensure their broad comparability between the US and the UK. Additional analyses using other measures, such as the University of California Los Angeles loneliness scales used only in USOC (Russel et al., 1978), produced consistent results. Third, further tests were conducted to explore whether the association between inter-household contact and mental well-being varied with socio-demographic characteristics such as solo living. No statistically significant moderating effects were found.

### Limitations and Future Research Directions

The limitations of this study suggest some important directions for further research. First, because inter-household contact was only measured once during the pandemic, the findings reveal only an association, not causality between inter-household contact and mental well-being. For example, it is possible that people who felt more isolated and lonelier tended to make virtual contact more frequently. To fully disentangle possible reverse causality, scholars would need to conduct in-depth and *in-situ* qualitative research to understand more fully the complex mechanisms underpinning the relationship between inter-household contact and older adults’ mental well-being. Second, due to data limitation and to ensure cross-national comparability, it was not possible to compare whether and how inter-household contact in various virtual forms (such as telephone calls vs. social media interactions) or with different people (such as family vs. friends) differentially related to mental well-being or its changes.

Third, the relatively small size of the samples, particularly in the US, meant that it was not possible to conduct sub-sample analysis, for example, by disaggregated age bands. Future research could rely on the analysis of larger samples to tease out further nuances in the relationship between inter-household contact and mental well-being across distinct demographic groups. Finally, although this study harmonised the US and UK data as much as it was possible for comparative analysis, the findings need to be carefully interpreted in the context of survey mode differences between the HRS and USOC. Coordinated cross-national efforts are needed to collect comparable data, for example with data collection using the same survey modes and the same question wordings. Such data will enable scholars to compare more closely the impact of the pandemic across different contexts.

## Discussion and Conclusions

This study is among the first to comparatively assess the association between inter-household contact and mental well-being in the context of the COVID-19 pandemic. Building on the analysis of national panel data from the US and the UK, the findings show that the pandemic has undermined older adults’ mental well-being, as evidenced by the notable increase in loneliness in the US and the decline in general mental well-being in the UK. While governments across a wide range of countries made efforts to protect older adults from COVID-19 infection and mortality (Lebow, 2020; Willem et al., 2021), the findings reported in this article contribute to the evidence base for policy development by suggesting that public health policymakers and practitioners should also address the looming mental health crisis cascading from the pandemic into this age group (Luchetti et al., 2020; Pierce et al., 2020).

In many countries, responses to COVID-19—at least before the rollout of mass vaccination programmes—centred on household-centred lockdown, shielding, and social distancing measures (Our World in Data, 2021). The findings in this study reveal the adverse impact of these measures on mental well-being and highlight inter-household interactions as a key resource to help sustain older adults’ mental well-being during crises such as the COVID-19 pandemic. Policymakers and practitioners need to take measures to pre-empt and mitigate the potential unintended implications of household-centred pandemic responses for mental well-being. Beyond the context of the pandemic, the findings also indicate the need to enable strong inter-household ties to bolster public mental health in the long run.

To help inform the development of policies and interventions, additional analyses were undertaken to explore how patterns of inter-household contact varied with various socio-demographic characteristics (full results are presented in the online supplementary file). In the US, men (vs. women) and those who had (vs. those who had not) contracted COVID-19 had less inter-household face-to-face contact during the pandemic. In both countries, ethnic/racial minority groups and those with poorer self-reported health also had less inter-household face-to-face contact during the pandemic. The explanation may be that racial/ethnic minority groups were less likely to have non-residential family members since they tended to live with extended family members (Nafilyan et al., 2021) and those with poorer health were more likely to have been required to practise household shielding. In the UK, women were more likely than men to rely on virtual-only inter-household contact during the pandemic. In the US, virtual-only inter-household contact was reported more frequently by those with a higher education degree, those who worked during the pandemic, and immigrants. Interestingly, in both countries, people living alone tended to engage in more inter-household face-to-face contact than people who lived with others during the pandemic. This may be because those living alone had a greater need for such contact or had built more durable inter-household ties. These supplementary results highlight specific socio-demographic groups whose inter-household contact was particularly affected during the pandemic. These insights will be crucial to developing targeted interventions.

The findings from the main study and additional analyses provide new and nuanced insights into how inter-household contact, in distinct face-to-face and virtual forms, was associated with older adults’ mental well-being. It is important to distinguish and compare the two forms of inter-household contact in the context of the COVID-19 pandemic, given that the pandemic severely curtailed face-to-face contact but increased virtual forms of contact. On the one hand, in line with the convoy and stress-coping theories, our findings suggest the mental health benefits associated with inter-household face-to-face contact. In both the US and the UK, more frequent face-to-face contact with non-residential family members and friends was associated with better general mental well-being and less loneliness during the pandemic. In both countries, inter-household face-to-face contact also seemed to have protected older adults from suffering a decline in general mental well-being and from becoming lonelier after the outbreak of the pandemic.

On the other hand, inter-household virtual contact appears to be associated with little mental health benefits for older adults during the pandemic. In both countries, virtual contact was not associated with older adults’ general mental well-being. Older adults with more frequent virtual contact were more likely to feel lonely during the pandemic in the US and to have become lonelier than before it in both countries. These results need to be interpreted with reference to the fact that the US respondents were surveyed using postal questionnaires, and the UK respondents completed their questionnaires online. Although the difference in survey mode means that the respondents who took part in the UK survey employed digital forms of communication, they did not benefit more from inter-household virtual contact than their US counterparts. Therefore, the results suggest the potentially limited role played by virtual contact in sustaining older adults’ mental well-being during the pandemic, regardless of national contexts.

The findings further highlight the importance of considering the interaction between inter-household face-to-face contact and virtual contact in understanding older adults’ mental well-being during the pandemic. Most notably, the findings show that, in both the US and the UK, those who enjoyed frequent inter-household contact in both face-to-face and virtual forms during the pandemic fared best in terms of general mental well-being in the US and in terms of loneliness in the UK. Virtual-only inter-household contact was negatively associated with mental well-being, potentially due to digital stress or burnout (Mheidly et al., 2020), but further research is required to unravel the complex causal mechanisms underlying this association. Nonetheless, the results clearly show that virtual interactions complement but cannot replace face-to-face interactions in helping sustain older adults’ mental well-being.

Last but not least, the findings suggest that, despite significant differences between the US and the UK, the relationship between inter-household contact and mental well-being is substantively similar. The findings also highlight the importance of inter-household contact, particularly face-to-face interactions, in helping sustain older adults’ general mental well-being and mitigate their loneliness in times of crisis. Thus, beyond formal material and care provision for older people, it is important to consider that “weak ties” and informal support maintained through inter-household interactions constitute a key social and health resource. As the importance of this resource cuts across the considerably different contexts in the US and the UK, it may also be generalisable to other countries. While inter-household contact was often taken for granted as a routine part of everyday life and non-residential care before the pandemic, its curtailment during the pandemic has brought its importance to the fore.

## Data Availability

Publicly available datasets were analysed in this study. The Health and Retirement Study (HRS) is a national longitudinal study of the economic, health, marital, and family status, as well as public and private support systems, of older Americans. The data were publicly released through the HRS website (https://hrs.isr.umich.edu/). Funding for the HRS is provided by the National Institute on Aging at NIH (U01 AG009740), with supplemental support from the Social Security Administration. The HRS is conducted by the Institute for Social Research (ISR) at the University of Michigan. The UK data were made available through the UK Data Archive. The UK Household Longitudinal Study (Understanding Society) is an initiative funded by the ESRC and various Government Departments and the Understanding Society COVID-19 Survey is funded by the UKRI, with scientific leadership by the ISER, University of Essex, and survey delivery by NatCen Social Research and Kantar Public.
