# Supplementary material for: COVID-19, Inter-household Contact and Mental Well-Being Among Older Adults in the US and the UK
Source: Front Sociol. 2021 Jul 26;6:714626. doi: 10.3389/fsoc.2021.714626 (PMC8350320; doi:10.3389/fsoc.2021.714626)
Supplement: Supplementary file 1 [file DataSheet1.docx]

Supplementary Material

**for**

**COVID-19, Inter-household Contact and Mental Well-being**

**among Older Adults in the US and the UK**

**Table S1.** Steps of sample construction.

**Table S2.** Models predicting general mental well-being during COVID-19 and its changes from before the pandemic (for Figure 4).

**Table S3.** Models predicting loneliness during COVID-19 and its changes from before the pandemic (for Figure 5).

**Table S4.** Models estimating the interaction of inter-household face-to-face and virtual contact in predicting general mental well-being during COVID-19 and its changes from before the pandemic (for Figure 6).

**Table S5.** Models estimating the interaction of inter-household face-to-face and virtual contact in predicting loneliness during COVID-19 and its changes from before the pandemic (for Figure 7).

**Table S6.** Models predicting inter-household face-to-face and virtual contact during COVID-19.

**Table S7.** Models predicting double exclusion from inter-household face-to-face and virtual contact and virtual-only inter-household contact during COVID-19.

| **Table S1.** Steps of sample construction | | | |
| --- | --- | --- | --- |
| **US** |  | **UK** |  |
| Steps of deletion | Remaining sample size | Steps of deletion | Remaining sample size |
| — | Full HRS COVID-19 early release v1 (Feb. 2021), *N* = 3,266, of whom 2,140 took part in the COVID-19 module | — | Full USOC COVID-19 survey Wave 3, *N* = 14,123 |
| Limit age range to 60 and above | *N* = 1,736 | Limit age range to 60 and above | *N* = 5,472 |
| Limit sample to respondents with valid (general mental well-being) records in prepandemic wave of HRS | *N* = 1,623 | Limit sample to respondents with valid (general mental well-being) records in prepandemic wave of USOC | *N* = 5,311 |
| Listwise deletion of missing values, following the order below:   - 90: face-to-face contact - 27: virtual contact - 28: general mental well-being during COVID-19 - 15: loneliness during COVID-19 - 18: changes in the level of loneliness before and during COVID-19 - 2: living alone - 31: working during COVID-19 - 2: self-reported health during COVID-19 - 19: satisfaction with household income | *N* = 1,391 respondents (observed both before and during COVID-19) | Listwise deletion of missing values, following the order below:   - 55: face-to-face contact - 8: virtual contact - 23: general mental well-being during COVID-19 - 1: Loneliness during COVID-19 - 3: changes in the level of loneliness before and during COVID-19 - 23: higher education degree - 3: self-reported health during COVID-19 - 14: ethnicity - 30: migrant status - 3: satisfaction with household income | *N* = 5,148 respondents (observed both before and during COVID-19) |
| *Notes*: HRS = Health and Retirement Survey. USOC = Understanding Society. | | | |

| **Table S2.** Models predicting general mental well-being during COVID-19 and its changes from before the pandemic (for Figure 4). | | | | |
| --- | --- | --- | --- | --- |
|  | M1: General mental well-being (standardised score, OLS linear regression, US) | M2: General mental well-being (standardised score, OLS linear regression, UK) | M3: General mental well-being changes (standardised score, OLS linear regression, US) | M4: General mental well-being changes (standardised score, OLS linear regression, UK) |
| Predictor | *B (SE)* | *B (SE)* | *B (SE)* | *B (SE)* |
| Inter-household contact during COVID-19 |  |  |  |  |
| Face-to-face contact | –0.075* | –0.079** | 0.046 | –0.094** |
| (high = more frequent) | (0.030) | (0.028) | (0.031) | (0.033) |
| Virtual contact | 0.000 | 0.024 | 0.024 | 0.029 |
| (high = more frequent) | (0.033) | (0.022) | (0.034) | (0.023) |
| Living alone (ref. = no) | 0.282*** | 0.319*** | 0.086 | 0.188* |
|  | (0.072) | (0.072) | (0.075) | (0.077) |
| Age | –0.000 | –0.004 | 0.008* | 0.005 |
|  | (0.004) | (0.005) | (0.004) | (0.005) |
| Female (ref. = male) | 0.141* | 0.236*** | –0.048 | 0.119* |
|  | (0.056) | (0.045) | (0.062) | (0.057) |
| Higher education degree (ref. = no) | –0.046 | 0.203*** | 0.080 | 0.092+ |
|  | (0.056) | (0.042) | (0.063) | (0.048) |
| Working during COVID-19 (ref. = no) | –0.131* | 0.158+ | –0.058 | 0.047 |
|  | (0.060) | (0.089) | (0.069) | (0.097) |
| Had/has COVID-19 (ref. = no) | 0.199 | 0.636* | 0.160 | 0.329+ |
|  | (0.199) | (0.251) | (0.144) | (0.175) |
| Ethnic/racial minority (ref. = no) | 0.035 | –0.316 | –0.020 | –0.358 |
|  | (0.072) | (0.225) | (0.078) | (0.408) |
| Migrant (ref. = no) | 0.078 | –0.115 | –0.025 | 0.108 |
|  | (0.090) | (0.113) | (0.113) | (0.182) |
| Self-reported health (high = better) | –0.299*** | –0.245*** | –0.090** | 0.071+ |
|  | (0.034) | (0.033) | (0.034) | (0.037) |
| Satisfaction with household income | –0.131*** | –0.143*** | 0.005 | 0.035 |
| (high = more satisfied) | (0.027) | (0.033) | (0.031) | (0.029) |
| Intercept | 1.150*** | 1.115** | –0.350 | –0.836* |
|  | (0.347) | (0.400) | (0.328) | (0.400) |
| *N* | 1,391 | 5,148 | 1,391 | 5,148 |
| *Notes*: OLS = Ordinary least squares. Ref. = Reference category. S.E. = standard errors, which were clustered at the household level to account for intra-household correlations. Weighted statistics with unweighted sample sizes.  + *p* < 0.10, * *p* < 0.05, ** *p* < 0.01, *** *p* < 0.001. | | | | |

| **Table S3.** Models predicting loneliness during COVID-19 and its changes from before the pandemic (for Figure 5). | | | | |
| --- | --- | --- | --- | --- |
|  | M1: Loneliness (ordinal logit regression, US) | M2: Loneliness (ordinal logit regression, UK) | M3: Changes in loneliness (multinomial logit regression, US) | M4: Changes in loneliness (multinomial logit regression, UK) |
| Predictor | *B (SE)* | *B (SE)* | *B (SE)* | *B (SE)* |
| ***Main models for the first two models, and predicting “less lonely” for the latter two models*** |  |  |  |  |
| Inter-household contact during COVID-19 |  |  |  |  |
| Face-to-face contact | –0.276*** | –0.031 | –0.230 | –0.013 |
| (high = more frequent) | (0.079) | (0.074) | (0.231) | (0.080) |
| Virtual contact | 0.190* | –0.102 | 0.234 | –0.078 |
| (high = more frequent) | (0.079) | (0.074) | (0.220) | (0.076) |
| Living alone (ref. = no) | 0.787*** | 1.540*** | 0.455 | 0.152 |
|  | (0.170) | (0.182) | (0.392) | (0.183) |
| Age | –0.000 | –0.027+ | –0.017 | –0.008 |
|  | (0.009) | (0.015) | (0.022) | (0.016) |
| Female (ref. = male) | 0.502** | 0.685*** | 0.098 | 0.254 |
|  | (0.152) | (0.160) | (0.358) | (0.155) |
| Higher education degree (ref. = no) | 0.294+ | –0.037 | –0.249 | –0.081 |
|  | (0.152) | (0.114) | (0.373) | (0.148) |
| Working during COVID-19 (ref. = no) | –0.364* | –0.004 | 0.298 | 0.158 |
|  | (0.177) | (0.166) | (0.419) | (0.236) |
| Had/has COVID-19 (ref. = no) | 0.439 | –0.197 | –1.454 | –0.004 |
|  | (0.493) | (0.281) | (1.063) | (0.489) |
| Ethnic/racial minority (ref. = no) | –0.381+ | –0.358 | 0.905* | 0.452 |
|  | (0.197) | (0.442) | (0.432) | (0.549) |
| Migrant (ref. = no) | –0.247 | –0.203 | –0.131 | 0.774* |
|  | (0.231) | (0.308) | (0.478) | (0.303) |
| Self-reported health (high = better) | –0.194* | –0.603*** | 0.054 | –0.269** |
|  | (0.083) | (0.086) | (0.195) | (0.084) |
| Satisfaction with household income | –0.318*** | –0.337*** | –0.133 | –0.084 |
| (high = more satisfied) | (0.082) | (0.073) | (0.232) | (0.072) |
| Intercept for M1 and M2 |  |  |  |  |
| Cut1 | –0.778 | –2.854** |  |  |
|  | (0.791) | (1.071) |  |  |
| Cut2 | 1.835* | –0.312 |  |  |
|  | (0.806) | (1.005) |  |  |
| Intercept for M3 and M4 |  |  | –1.721 | –0.354 |
|  |  |  | (1.562) | (1.088) |
| ***Predicting “more lonely” for the latter two models*** |  |  |  |  |
| Inter-household contact during COVID-19 |  |  |  |  |
| Face-to-face contact |  |  | –0.241** | –0.085 |
| (high = more frequent) |  |  | (0.088) | (0.079) |
| Virtual contact |  |  | 0.227** | 0.160* |
| (high = more frequent) |  |  | (0.085) | (0.077) |
| Living alone (ref. = no) |  |  | 0.460* | 0.700*** |
|  |  |  | (0.187) | (0.175) |
| Age |  |  | –0.019+ | –0.023+ |
|  |  |  | (0.011) | (0.013) |
| Female (ref. = male) |  |  | 0.571*** | 0.593*** |
|  |  |  | (0.172) | (0.153) |
| Higher education degree (ref. = no) |  |  | 0.344* | 0.033 |
|  |  |  | (0.172) | (0.131) |
| Working during COVID-19 (ref. = no) |  |  | –0.270 | –0.013 |
|  |  |  | (0.191) | (0.179) |
| Had/has COVID-19 (ref. = no) |  |  | –0.054 | –0.502 |
|  |  |  | (0.512) | (0.560) |
| Ethnic/racial minority (ref. = no) |  |  | –0.325 | –0.455 |
|  |  |  | (0.213) | (0.582) |
| Migrant (ref. = no) |  |  | –0.492 | 0.539+ |
|  |  |  | (0.314) | (0.305) |
| Self-reported health (high = better) |  |  | 0.087 | –0.396*** |
|  |  |  | (0.091) | (0.091) |
| Satisfaction with household income |  |  | –0.291*** | 0.052 |
| (high = more satisfied) |  |  | (0.078) | (0.084) |
| Intercept |  |  | 0.535 | 0.130 |
|  |  |  | (0.871) | (0.982) |
| *N* | 1,391 | 5,148 | 1,391 | 5,148 |
| *Notes*: OLS = Ordinary least squares. Ref. = Reference category. S.E. = standard errors, which were clustered at the household level to account for intra-household correlations. Weighted statistics with unweighted sample sizes.  + *p* < 0.10, * *p* < 0.05, ** *p* < 0.01, *** *p* < 0.001. | | | | |

| **Table S4.** Models estimating the interaction of inter-household face-to-face and virtual contact in predicting general mental well-being during COVID-19 and its changes from before the pandemic (for Figure 6). | | | | |
| --- | --- | --- | --- | --- |
|  | M1: General mental well-being (standardised score, OLS linear regression, US) | M2: General mental well-being (standardised score, OLS linear regression, UK) | M3: General mental well-being changes (standardised score, OLS linear regression, US) | M4: General mental well-being changes (standardised score, OLS linear regression, UK) |
| Predictor | *B (SE)* | *B (SE)* | *B (SE)* | *B (SE)* |
| Inter-household contact during COVID-19 |  |  |  |  |
| Face-to-face contact | –0.078** | –0.079** | 0.039 | –0.094** |
| (high = more frequent) | (0.030) | (0.028) | (0.030) | (0.033) |
| Virtual contact | 0.004 | 0.024 | 0.033 | 0.029 |
| (high = more frequent) | (0.034) | (0.021) | (0.033) | (0.023) |
| Face-to-face × virtual | –0.023 | 0.003 | –0.059+ | –0.003 |
|  | (0.030) | (0.028) | (0.030) | (0.027) |
| Living alone (ref. = no) | 0.280*** | 0.319*** | 0.081 | 0.188* |
|  | (0.072) | (0.072) | (0.075) | (0.077) |
| Age | –0.000 | –0.003 | 0.008* | 0.005 |
|  | (0.004) | (0.005) | (0.004) | (0.005) |
| Female (ref. = male) | 0.139* | 0.236*** | –0.052 | 0.118* |
|  | (0.056) | (0.045) | (0.063) | (0.057) |
| Higher education degree (ref. = no) | –0.046 | 0.203*** | 0.081 | 0.092+ |
|  | (0.056) | (0.042) | (0.063) | (0.048) |
| Working during COVID-19 (ref. = no) | –0.132* | 0.158+ | –0.061 | 0.047 |
|  | (0.060) | (0.091) | (0.069) | (0.099) |
| Had/has COVID-19 (ref. = no) | 0.194 | 0.637* | 0.148 | 0.329+ |
|  | (0.197) | (0.251) | (0.142) | (0.174) |
| Ethnic/racial minority (ref. = no) | 0.035 | –0.316 | –0.020 | –0.358 |
|  | (0.071) | (0.225) | (0.077) | (0.408) |
| Migrant (ref. = no) | 0.072 | –0.116 | –0.039 | 0.108 |
|  | (0.090) | (0.113) | (0.112) | (0.183) |
| Self-reported health (high = better) | –0.300*** | –0.245*** | –0.093** | 0.071+ |
|  | (0.034) | (0.033) | (0.033) | (0.037) |
| Satisfaction with household income | –0.132*** | –0.143*** | 0.004 | 0.035 |
| (high = more satisfied) | (0.027) | (0.033) | (0.030) | (0.029) |
| Intercept | 1.164*** | 1.112** | –0.315 | –0.833* |
|  | (0.348) | (0.411) | (0.326) | (0.407) |
| *N* | 1,391 | 5,148 | 1,391 | 5,148 |
| *Notes*: Ref. = Reference category. S.E. = standard errors, which were clustered at the household level to account for intra-household correlations. Weighted statistics with unweighted sample sizes.  + *p* < 0.10, * *p* < 0.05, ** *p* < 0.01, *** *p* < 0.001. | | | | |

| **Table S5.** Models estimating the interaction between inter-household face-to-face and virtual contact in predicting loneliness during COVID-19 and its changes from before the pandemic (for Figure 7). | | | | |
| --- | --- | --- | --- | --- |
|  | M1: Loneliness (ordinal logit regression, US) | M2: Loneliness (ordinal logit regression, UK) | M3: Changes in loneliness (multinomial logit regression, US) | M4: Changes in loneliness (multinomial logit regression, UK) |
| Predictor | *B (SE)* | *B (SE)* | *B (SE)* | *B (SE)* |
| ***Main models for the first two models, and predicting “less lonely” for the latter two models*** |  |  |  |  |
| Inter-household contact during COVID-19 |  |  |  |  |
| Face-to-face contact | –0.279*** | –0.039 | –0.237 | 0.001 |
| (high = more frequent) | (0.078) | (0.070) | (0.237) | (0.078) |
| Virtual contact | 0.196* | –0.121 | 0.239 | –0.053 |
| (high = more frequent) | (0.080) | (0.078) | (0.225) | (0.079) |
| Face-to-face × virtual | –0.043 | –0.100 | 0.091 | 0.122 |
|  | (0.071) | (0.069) | (0.223) | (0.080) |
| Living alone (ref. = no) | 0.783*** | 1.548*** | 0.468 | 0.143 |
|  | (0.171) | (0.178) | (0.390) | (0.183) |
| Age | –0.000 | –0.029* | –0.017 | –0.006 |
|  | (0.009) | (0.014) | (0.022) | (0.016) |
| Female (ref. = male) | 0.499** | 0.679*** | 0.104 | 0.266+ |
|  | (0.152) | (0.158) | (0.352) | (0.154) |
| Higher education degree (ref. = no) | 0.295+ | –0.033 | –0.256 | –0.083 |
|  | (0.152) | (0.114) | (0.375) | (0.148) |
| Working during COVID-19 (ref. = no) | –0.367* | –0.025 | 0.309 | 0.177 |
|  | (0.177) | (0.157) | (0.421) | (0.235) |
| Had/has COVID-19 (ref. = no) | 0.432 | –0.222 | –1.426 | 0.027 |
|  | (0.494) | (0.282) | (1.062) | (0.484) |
| Ethnic/racial minority (ref. = no) | –0.382+ | –0.352 | 0.904* | 0.460 |
|  | (0.198) | (0.451) | (0.427) | (0.557) |
| Migrant (ref. = no) | –0.256 | –0.197 | –0.099 | 0.758* |
|  | (0.231) | (0.311) | (0.460) | (0.307) |
| Self-reported health (high = better) | –0.196* | –0.598*** | 0.058 | –0.275** |
|  | (0.083) | (0.086) | (0.198) | (0.084) |
| Satisfaction with household income | –0.320*** | –0.339*** | –0.132 | –0.082 |
| (high = more satisfied) | (0.082) | (0.074) | (0.229) | (0.072) |
| Intercept for M1 and M2 |  |  |  |  |
| Cut1 | –0.804 | –2.996** |  |  |
|  | (0.791) | (1.042) |  |  |
| Cut2 | 1.809* | –0.449 |  |  |
|  | (0.806) | (0.980) |  |  |
| Intercept for M3 and M4 |  |  | –1.788 | –0.533 |
|  |  |  | (1.592) | (1.119) |
| ***Predicting “more lonely” for the latter two models*** |  |  |  |  |
| Inter-household contact during COVID-19 |  |  |  |  |
| Face-to-face contact |  |  | –0.241** | –0.080 |
| (high = more frequent) |  |  | (0.088) | (0.080) |
| Virtual contact |  |  | 0.228** | 0.158* |
| (high = more frequent) |  |  | (0.085) | (0.078) |
| Face-to-face × virtual |  |  | –0.011 | 0.002 |
|  |  |  | (0.076) | (0.070) |
| Living alone (ref. = no) |  |  | 0.459* | 0.701*** |
|  |  |  | (0.187) | (0.175) |
| Age |  |  | –0.019+ | –0.023+ |
|  |  |  | (0.011) | (0.013) |
| Female (ref. = male) |  |  | 0.571*** | 0.593*** |
|  |  |  | (0.172) | (0.153) |
| Higher education degree (ref. = no) |  |  | 0.345* | 0.033 |
|  |  |  | (0.172) | (0.131) |
| Working during COVID-19 (ref. = no) |  |  | –0.271 | –0.012 |
|  |  |  | (0.191) | (0.177) |
| Had/has COVID-19 (ref. = no) |  |  | –0.056 | –0.500 |
|  |  |  | (0.513) | (0.559) |
| Ethnic/racial minority (ref. = no) |  |  | –0.326 | –0.458 |
|  |  |  | (0.213) | (0.582) |
| Migrant (ref. = no) |  |  | –0.496 | 0.541+ |
|  |  |  | (0.317) | (0.306) |
| Self-reported health (high = better) |  |  | 0.086 | –0.396*** |
|  |  |  | (0.091) | (0.091) |
| Satisfaction with household income |  |  | –0.291*** | 0.053 |
| (high = more satisfied) |  |  | (0.078) | (0.084) |
| Intercept |  |  | 0.542 | 0.129 |
|  |  |  | (0.870) | (0.974) |
| *N* | 1,391 | 5,148 | 1,391 | 5,148 |
| *Notes*: Ref. = Reference category. S.E. = standard errors, which were clustered at the household level to account for intra-household correlations. Weighted statistics with unweighted sample sizes.  + *p* < 0.10, * *p* < 0.05, ** *p* < 0.01, *** *p* < 0.001. | | | | |

| **Table S6.** Models predicting inter-household face-to-face and virtual contact during the pandemic. | | | | |
| --- | --- | --- | --- | --- |
|  | M1: Face-to-face contact (OLS linear regression, US) | M2: Face-to-face contact (OLS linear regression, UK) | M3: Virtual contact (OLS linear regression, US) | M4: Virtual contact (OLS linear regression, UK) |
| Predictor | *B (SE)* | *B (SE)* | *B (SE)* | *B (SE)* |
| Living alone (ref. = no) | 0.125+ | 0.151* | 0.014 | 0.219*** |
|  | (0.073) | (0.071) | (0.079) | (0.066) |
| Age | 0.001 | 0.015** | –0.023*** | –0.033*** |
|  | (0.005) | (0.005) | (0.004) | (0.005) |
| Female (ref. = male) | 0.200*** | 0.072 | 0.466*** | 0.476*** |
|  | (0.059) | (0.052) | (0.064) | (0.056) |
| Higher education degree (ref. = no) | –0.088 | 0.031 | 0.201** | 0.116** |
|  | (0.071) | (0.045) | (0.068) | (0.045) |
| Working during COVID-19 (ref. = no) | 0.089 | –0.021 | 0.052 | –0.227*** |
|  | (0.082) | (0.073) | (0.086) | (0.063) |
| Had/has COVID-19 (ref. = no) | –0.329+ | –0.143 | –0.193 | 0.192+ |
|  | (0.177) | (0.222) | (0.152) | (0.112) |
| Ethnic/racial minority (ref. = no) | –0.209* | –0.279* | –0.045 | –0.387+ |
|  | (0.094) | (0.142) | (0.092) | (0.209) |
| Migrant (ref. = no) | –0.156 | –0.125 | 0.180 | 0.217+ |
|  | (0.125) | (0.100) | (0.120) | (0.111) |
| Self-reported health (high = better) | 0.077+ | 0.120*** | 0.128*** | 0.115*** |
|  | (0.040) | (0.033) | (0.039) | (0.029) |
| Satisfaction with household income | 0.073* | 0.022 | 0.054 | –0.010 |
| (high = more satisfied) | (0.036) | (0.029) | (0.036) | (0.034) |
| Intercept | –0.526 | –1.579*** | 0.709* | 1.632*** |
|  | (0.387) | (0.356) | (0.360) | (0.384) |
| *N* | 1,391 | 5,148 | 1,391 | 5,148 |
| *Note*: Ref. = Reference category. S.E. = standard errors, which were clustered at the household level to account for intra-household correlations. Dependent variables are the standardised scores for face-to-face and virtual inter-household contact. Weighted statistics with unweighted sample sizes.  + *p* < 0.10, * *p* < 0.05, ** *p* < 0.01, *** *p* < 0.001. | | | | |

| **Table S7.** Models predicting double exclusion from inter-household face-to-face and virtual contact and virtual-only inter-household contact during the COVID-19 pandemic. | | | | |
| --- | --- | --- | --- | --- |
|  | M1: Double exclusion from both face-to-face and virtual contact (logit regression, US) | M2: Double exclusion from both face-to-face and virtual contact (logit regression, UK) | M3: Virtual-only contact (logit regression, US) | M4: Virtua-only contact (logit regression, UK) |
| Predictor | *B (SE)* | *B (SE)* | *B (SE)* | *B (SE)* |
| Living alone (ref. = no) | –0.490+ | –0.346 | 0.156 | –0.214 |
|  | (0.266) | (0.228) | (0.438) | (0.223) |
| Age | 0.030* | 0.021+ | –0.015 | –0.035+ |
|  | (0.015) | (0.012) | (0.022) | (0.021) |
| Female (ref. = male) | –0.904*** | –0.989*** | 0.515 | 0.693** |
|  | (0.224) | (0.146) | (0.414) | (0.232) |
| Higher education degree (ref. = no) | –0.497+ | –0.299* | –0.988* | –0.186 |
|  | (0.256) | (0.138) | (0.468) | (0.174) |
| Working during COVID-19 (ref. = no) | –0.041 | 0.214 | 0.775* | –0.230 |
|  | (0.286) | (0.206) | (0.382) | (0.216) |
| Had/has COVID-19 (ref. = no) | 0.801 | –1.086+ | –0.896 | 0.670 |
|  | (0.622) | (0.574) | (1.056) | (0.459) |
| Ethnic/racial minority (ref. = no) | 0.448 | 1.359+ | 0.695 | –0.614 |
|  | (0.319) | (0.821) | (0.449) | (0.574) |
| Migrant (ref. = no) | –0.825+ | –0.385 | 1.178** | –0.112 |
|  | (0.467) | (0.589) | (0.437) | (0.301) |
| Self-reported health (high = better) | –0.288* | –0.269*** | –0.022 | –0.191 |
|  | (0.119) | (0.082) | (0.173) | (0.153) |
| Satisfaction with household income | –0.168 | –0.002 | 0.011 | 0.022 |
| (high = more satisfied) | (0.121) | (0.091) | (0.146) | (0.140) |
| Intercept | –2.257+ | –1.970* | –2.985+ | 0.099 |
|  | (1.206) | (0.936) | (1.544) | (1.438) |
| *N* | 1,391 | 5,148 | 1,391 | 5,148 |
| *Note*: Ref. = Reference category. S.E. = standard errors, which were clustered at the household level to account for intra-household correlations. Double exclusion refers to respondents falling in the bottom quartiles (25%) of both face-to-face and virtual inter-household contact in their country. Virtual-only contact refers to respondents falling in the bottom quartile of face-to-face contact but top quartile of virtual inter-household contact in their country. Weighted statistics with unweighted sample sizes.  + *p* < 0.10, * *p* < 0.05, ** *p* < 0.01, *** *p* < 0.001. | | | | |
